# Cutting the Nerves

**Published:** 1870-01

**Authors:** 


### CUTTING THE NERVES.

BY P. H. H.

In health the eye delights in seeing and the ear in hearing, and we are not conscious of eye or ear as such. So every sense, every organ, every movement is intended by nature to bring into the commonwealth of life its little income of pleasure, and make us only conscious of the pleasure.

Thus the digestion of food, the beating of the heart, the movements of the blood, the breathing by the lungs, the nourishment of the body, the pouring out of the secretions, the warming of the body by its own vital chemistry, all go on in health without consciousness and of course, without a thought or care. What a mystery is this that all these actions, some of them instantly essential to life, should go on whether we are awake or asleep, and from infancy to age with almost perfect rhythmical regularity, and never so long as in perfect health, compel from us the least attention. The nerves which control and take care of those operations act constantly from and in harmony with guiding impressions, and only when things go wrong do they send to the brain the warning and outcry of pain, thus compelling attention, and suspending to a greater or less degree the natural use and employment of the part.

Look out upon the street and observe how light and elastic some of the horses move as they go by, and then see others go crippling and flinching over the pavement, their drivers being scarcely able to force them into a hobbling trot. What is the matter with these lame horses? Ask the owner and he will tell you they have been *foundered*; that is driven hard and fed or watered when fatigued and heated, and inflammation has settled in the limbs, but especially in the soft elastic cushion of capillary blood vessels and nerves which is the growing point or matrix of the hoof.

When I was a medical student my preceptor had a fine bay horse that he drove in his practice, but sometimes at great disadvantage from his having been foundered. He finally determined to cut the nerves of sensation which come from the matrix of the hoof on each side of the fore-arm and just above the fetlock joint. The operation was done, the horse got up and moved off as though he had never heard of such a thing as being *foundered!* More than twenty horses were operated on in this manner in that vicinity, and every horse without exception lost the hoof by inflamation and sloughing, the time varying with the more or less hard use of the horse upon the road.

To-day a woman came to me saying, "Doctor I can't see with my left eye and my right pains me if I only use it three minutes." Facts are, the woman has worked on black stuffs by lamplight, disregarding the monition of pain, until she has cut the optic nerve—paralysed it on the left side.

Sometimes when the nerves cry out with hard pain and the doctors don't know how to cure or can't cure the *cause* of the pain, they give them morphine, this cuts the nerve and the brain cannot *feel the pain.*

---

HERALDED INTO THE WORLD.—The Germans announce bethrothals in the papers as regularly as we do marriages and deaths. They herald births with much flourish. For instance: I have the honour politely to announce to my relations and friends the happy delivery of my dear wife, Julia, maiden name, Satmarn, of a healthy, strong boy. Cologne, August 17, 1869, Edward Fomm."

